# Dental Germicides

**Published:** 1903-08-15

**Authors:** E. Mawhinney


### DENTAL GERMICIDES.

BY E. MAWHINNEY, D.D.S.

The following abstract of a paper read before the Chicago Dental Society will throw some new light on the relative values of some old and introduce some new or little known dental germicides.

Not much longer will it do to treat all conditions with certain remedies, simply because our fathers did or even because we have found that in previous cases good results have followed their use. Our literature is full of conflicting statements in regard to the potency of various agents classed as germicides, because no absolutely reliable tests have ever been made to determine their relative values. Each investigator has used a different method, a different germ, or so modified his research that necessarily different results have been attained.

In making experimental tests, it is essential that the agent used be pure and reliable; that the germs be exposed to it in equal numbers under the same conditions; that they be at their maximum height of virulency, should be pure cultures, and that they be cultivated in media and temperature most favorable to their growth. In the experiments from which the following table is made up the following method was used: Organisms were grown in bouillon made from lean beef (not beef extract), in the usual manner, and neutralized with sodium hydrate (not sodium bicarbonate). In series D and E it was made slightly alkaline. The germs were grown and distributed throughout the media in equal numbers, as shown by microscopic examination. The germs were transferred in loopfuls to small squares (a centimeter) of filter paper, which was previously sterilized and kept in a petri dish; there they were allowed to dry; then on to this was carried by means of the loop sufficient of the medicament to completely cover the filter paper, and left for various lengths of time, when each square was washed, so as to remove the medicament, and planted in fresh tubes of culture media, and placed in an incubator, a 37 deg. centigrade. Readings were taken from time to time for a week. The germicidal power of the medicaments is here determined by the *time* necessary to expose germs to it, and, as you will see, a great difference appears. You will notice that some agents were used in full strength and others in percent solutions, according as they could be used in practice.

In all of these series of experiments I began by exposing the germ to the medicament five minutes, and worked each way from that point, according as growth appeared or not.

When doubt existed, inoculations were made in fresh media and in animals—guinea pigs and young rabbits mostly.

In this table only final results are given. They are made up after many repetitions, and six different germs were used. The variations in time were probably due to the resistance of the different germs.

| Agent. | Percent, Solution. | Time Required, Minutes. |
|---|---|---|
| Oil cassia | Full strength | 35 to 60 |
| Oil cinnamon | Full strength | 35 to 60 |
| Oil cloves | Full strength | 35 to 60 |
| Eugenol | Full strength | 32 |
| Oil cajeput | Full strength | 40 to 55 |
| Oil eucalyptus | Full strength | 40 to 60 |
| Oil wintergreen | Full strength | 40 to 60 |
| Oil peppermint | Full strength | 30 to 55 |
| Oil pennyroyal | Full strength | 35 to 45 |
| Permanganate of potash | 10 percent strength | 25 to 25 |
| Carbolic acid | Full strength | 10 to 30 |
| Creosote | Full strength | 15 to 40 |
| Phecene | Sat sol | 10 to 12 |
| Trikresol | Full strength | 2 to 5 |
| Kresamin | Full strength | 2 to 5 |
| Bichloride of mercury | 1-1,000 | 5 to 25 |
| Sublamine | 1-500 | 2 to 5 |
| Phenol sulphonic | Full strength | 2 to 5 |
| Chinosol | 10 percent | 1 |
| Formalin | Full strength | 1 to 3 |
| Creolin | Full strength | 3 to 8 |

This table only shows the time required to completely destroy all life. Nearly all agents showed marked restraint in less time. Many of the germs exposed to the essential oils fifteen, and indeed thirty minutes grew as quickly and as luxuriantly as the controller.

You will note the excellent showing made by the following agents: formalin, sublamine, phenol-sulphonic acid, trikresol, creolin, kresamin, phecene, chinosol.

The application of germicides as such to treatment of oral diseases is quite limited; it is only in violent, acute, chronic, necrotic suppurations; in syphilitic ulcers, eczema, etc., and in such cases the selection of the particular agent will be determined by the conditions present. They are also of value as hand and instrument disinfectors.

Formalin is a colorless liquid, resembling water in appearance, and is a forty percent solution of formaldehyde gas. It is probably the most potent germicide that can be used. Its dental uses are limited, because of its extreme irritating property. I have used it in old chronic abscesses, but in nearly every instance severe pain and swelling resulted which has caused me to abandon it except in weak dilutions in such agents as creosote.

Paraform, a new solid polymer, has recently been recommended. There is a class of cases where it is of value, if used with care. I refer to old blind abscesses on the roots of teeth, containing small tortuous canals. This agent readily gives up formaldehyde gas, which is very penetrating. It should only be placed in the large entrance to the pulp chamber, and not down in the root-canals, and even then it stirs up some irritation. In coming to this conclusion, I have lost some teeth from its use, but if you are careful and use it as stated you will find it of excellent value. Recently some practitioners have recommended it as a component part of root-fillings. I am somewhat skeptical of the result. In old chronic cases, where there is little or no discharge of pus, but instead a thin, ichorous fluid comes weeping down into the canal, cases that are not causing any great amount of pain, but sore and constantly annoying, in all such cases I get good results from this drug. It always increases the soreness and inflammation, which soon terminates in resolution. Perhaps the most valuable use we can make of this agent is as a disinfectant for foul rooms, for operating rooms, where serious surgical cases are attended to, for instruments, especially those used on syphilitic cases.

Paraform has recently been put upon the market in tablet form, especially designed for use in Schering's sterilizer. It is both effective and convenient.

Bichloride of mercury, as a germicide, was first brought to the attention of the medical profession by Koch, since which time its use has become almost universal. It is a potent germicide toward all germs that have a very permeable cell wall. It is very corrosive, producing insoluble coagulum, and therefore limiting its power. Its most serious objections are its irritant and toxic properties. In dental practice its use has been quite generally abandoned, except as a hand disinfectant, and on gauze for packing a suppurating antrum, also in syphilitic cases.

Sublamine: Ethyenediamine, sulphate of mercury. A new agent, recommended as a substitute for bichloride of mercury.

Ethylenediamine is an organic base, with a chemical formula of $CH_2—NH_2$

$$\begin{array}{l} CH_2—NH_2 \\ | \\ CH_2—NH_2 \end{array}$$

It is a clear, colorless liquid of alkaline reaction, and gives off the odor of ammonia. This substance is used in connection with several coagulant germicides, for the purpose of reducing their irritant properties and increasing their penetrative power. Sublamine comes to us in solid form, and is freely soluble in water. I have been using it in the strength of 1 in 500, and find it but very slightly irritating. It is a non-coagulant, and will penetrate much more deeply than bichloride. In all the tests it has proven much more efficacious than bichloride, and certainly is much more agreeable to use. I can most heartily recommend it for sterilizing hands, washing indolent ulcers, flushing the antrum for washing through chronic abscesses, sterilizing the skin before operations. For all these purposes I have been using it in my private practice, as well as in the public infirmary. It is a chemical germicide, carrying pus into solution.

Phenol-sulphonic acid is a light, reddish-colored liquid, made by combining equal parts of sulphuric and carbolic acid. It is not so coagulant or irritating as either of the substances from which it is made. It can be used in full strength for burning through old chronic abscesses, and is especially recommended where cases are of long standing, with more or less of bone absorption around the apex of the root. It is valuable to enlarge root-canals, and to burn out the socket after a badly-abscessed tooth is removed. Of course, it must always be used with caution. A fifty percent solution is especially recommended to aid in the exfoliation of necrosed bone; it will also disintegrate and dissolve small pieces of tooth and necrosed bone that may be left after burring or curetting about the jaws. I use it on gauze for the first packing after such operations, especially in the antrum. Weaker solutions may be used to wash out after surgical operations on the jaws. I have come to look upon it as one of my most valuable agents.

Trikresol; another product from Schering's chemical laboratory. Its compositions is as follows: Ortho-cresol, thirty-five percent; meta-cresol, forty percent, and para-cresol, forty percent. It is a clear liquid of pungent odor, resembling phenol; turns slightly red on exposure to strong sunlight. It is soluble in two percent water, but freely in alcohol and oils. It is a splendid germicide, as shown by these experiments, and an agreeable preparation to use. I have been using it for four years, and now find I am using it in almost every condition where I formerly used carbolic acid or creosote. It is not so escharotic as carbolic acid; will penetrate much deeper into vegetable cells, and will destroy spores. Two percent solution is antiseptic. I recommend it to burn out old abscesses; as a dressing in root-canals in acute apical pericementitis; in putrescent pulp; to relieve odontalgia, applied warm or almost hot. It penetrates the dentine as readily as the essential oils, but does not discolor it. A little (not an excess) is useful as a dressing after pulps are extirpated, before filling root-canals. To keep scalers and such instruments sterile while using, I keep them in a ten percent solution in alcohol and water. It is an excellent agent, used full strength, as a first treatment to pus pockets about the roots of teeth.

Kresamin is the name given to a combination of ethyenediamine and trikresol, containing equal parts of each. It is a reddish-colored, phenol-like liquid; has an agreeable odor, and is very slightly irritant. It is practically non-caustic, and but feebly coagulant. It is powerfully germicidal, equal to 1 in 500 bichloride of mercury. I have been using it lately in the clinic, with most flattering results. I am satisfied if used in acute or recent chronic abscesses it will be of value. I wash through such abscesses freely with it. I have passed some around among a few of my dental friends, and they are all delighted with the results they are getting with it. It is freely soluble, and may be used as an antiseptic in dilute solutions. It is a chemical disinfectant. When brought in contact with thick pus, kresamin seems to immediately dissolve it, and turn it a dark brown color. In apical pericementitis from any cause, it seems to be of great value; also applied to inflamed pulps it has an immediate quieting influence. In all inflammations accompanied with pus formation I am sure of its efficacy.

Chinosol has a chemical formula of $C_9$ $H_6$ N. $KSO_4$, and is prepared by the action of sulphate of potassium on chinoline, a basic coal-tar derivatice. It occurs in the form of a crytalline yellow powder, possessing a very slight odor, and pungent coal-tar taste. It is freely soluble in water, but is insoluble in ether or alcohol. It is a chemical germicide. When brought in contact with pus in the slightly alkaline fluid of the tissues, it is readily broken up into oxychlorine, and it is this that is so powerfully germicidal. So far as my knowledge goes, it is the most potent germicide, so far as pus germs are concerned, of any agent at our command. I began using and recommending it for the eradication of pus in 1893, since which time it is my main standby. It has two slight objections, namely, it corrodes steel instruments (not others), and has a slight tendency to darken teeth; such discoloration is very readily removed with any oxygen bleacher. Being almost devoid of odor. it is not a very good deodorant for foul-smelling dentine, but as a pus-destroyer, it certainly has no equal.

It is practically non-irritating; is wholly non-coagulant, and non-caustic. I used it in two percent solution for washing out bad pus pockets, abscesses in antrum, and alveolus. I use a ten percent solution in chronic, foul, violent abscesses, and all other violent suppurations. It is used only for the purpose of getting rid of pus. When you need to burn out necrotic tissue, it is not recommended. It is wholly non-toxic, and can be used *ad libitum* in these solutions. Injected into a forming abscess, boil or carbuncle, it will immediately get rid of the pus. In any case of violent pus infection, where there is danger of serious results, this is the most efficient agent, especially in streptococcic infection which is active you can use no better drug. Chinosol gauze, absorbent cotton and soap may be had in the market. If you have an abscessed antrum, where pus is rapidly being formed, try chinosol irrigation and chinosol gauze pack to control it, and you will be delighted.—*Dental Review.*

